# Survey data on public perceptions towards flying cars and flying taxi services

**DOI:** 10.1016/j.dib.2022.107981

**Published:** 2022-02-22

**Authors:** Ugur Eker, Grigorios Fountas, Sheikh Shahriar Ahmed, Panagiotis Ch. Anastasopoulos

**Affiliations:** a Turkish Airlines, Istanbul, Turkey; b Transport Research Institute, School of Engineering and the Built Environment, Edinburgh Napier University, Edinburgh, United Kingdom; c Department of Civil, Structural and Environmental Engineering, Stephen Still Institute for Sustainable Transportation and Logistics, University at Buffalo – The State University of New York, Buffalo, NY, United States

**Keywords:** Flying car, Flying taxi, Urban air mobility, Advanced air mobility, Public perception, Willingness to use, Willingness to pay

## Abstract

An online survey was conducted to evaluate public perceptions towards an emerging transportation technology, namely the flying car, which is expected to join the existing traffic fleet within the following decades. Responses from 692 survey participants were collected. Approximately 84% of the participants were from the United States, and the remaining 16% were from the rest of the world. The data resulting from the survey include several aspects of public perceptions towards flying cars, as for example: willingness to use and pay for flying cars; willingness to use and pay for flying taxi services; perceptions towards potential benefits and concerns arising from the future use of flying cars; perceptions towards considering residence relocation; and perceptions towards potential security measures to improve operational safety of flying cars. In addition, information relating to several dimensions of driving and travel behaviours and habits, and socio-demographic information of the participants were also collected. The dataset can be used as a baseline to design future surveys on Advanced Air Mobility (AAM) and flying cars, and to compare consumer perceptions across different regions and during different time periods.

## Specifications Table

**Table:** 

Subject	Transportation
Specific subject area	Flying Car; Flying Taxi; Public Perceptions; Willingness to Use; Willingness to Pay
Type of data	CSV file, Text (data dictionary).
How the data were acquired	SurveyMonkey
Data format	Raw, Processed.
Description of data collection	The survey took place in March 2017. It was distributed by 35 individuals through 35 unique distributor-specific links. The collected individual responses were then integrated into a single file for recoding and subsequent analysis.
Data source location	University at Buffalo – The State University of New York, United States.
Data accessibility	Repository name: Mendeley Data Data identification number: 10.17632/276ztbs7jc.1 Direct URL to data: https://dx.doi.org/10.17632/276ztbs7jc.1
Related research article	Eker, U., Fountas, G., & Anastasopoulos, P. C. (2020). An exploratory empirical analysis of willingness to pay for and use flying cars. *Aerospace Science and Technology, 104*, 105,993. DOI: https://doi.org/10.1016/j.ast.2020.105993

## Value of the Data

• The dataset provides one of the earliest insights into public perception towards flying car technologies. To the best of the author's knowledge, this survey was the first of its kind. As public perception towards this emerging technology continues to evolve, the insights offered by this dataset can serve as a benchmark for future studies to draw comparison and track the direction of evolution.
• The dataset can serve as a baseline for designing or comparing future studies on flying cars and Advanced Air Mobility (AAM), as well as other emerging transportation technologies. As additional information regarding flying cars and AAM technologies become available to the public, future surveys can incorporate the up-to-date information (e.g., purchase cost, cost of a ride in ridesharing framework, safety and security features offered). Furthermore, to derive policy recommendations, questions focused on evaluating the potential effectiveness of the most recent technical breakthroughs (AAM traffic management systems, operational noise level, redundancy offered by the AAM vehicle for enhanced safety) can be added.
• The dataset may be analysed using several methodological approaches (e.g., statistical and econometric methods, machine learning, and deep learning algorithms).
• This dataset can be leveraged by professors and educators to illustrate the use of statistical and econometric methods to analyse survey-collected data.

## 1 Data Description

A brief overview of the topics covered in the survey questionnaire is presented in Table 1.

**Table 1:** List and brief description of topics covered in the survey.

Topic	Description	Measure
Familiarity with advanced vehicle safety features	Level of familiarity and ownership of vehicles with advanced vehicle safety features (i.e., emergency automatic braking, lane keeping assist, adaptive cruise control, left turn assist, adaptive headlights, blind-spot monitoring)	Level of familiarity: 4-point Likert scale - very unfamiliar to very familiar; Ownership: Yes, No.
Ownership and usage scenarios of Flying cars	Willingness to own and operate a flying car if operated: • Manually, • Autonomously. Willingness to rent and operate a flying car if operated: • Manually, • Autonomously. Hire a flying taxi service/ridesharing service if operated: • By a human pilot, • Autonomously.	4-point Likert scale - very unlikely to very likely.
Willingness to purchase Flying cars	Willingness to purchase a flying car at varying pricing scenarios.	4-point Likert scale - very unlikely to very likely.
Willingness to pay for Flying car based ridesharing service	Willingness to pay for a ride in flying car based ridesharing service at varying pricing scenarios.	4-point Likert scale - very unlikely to very likely.
Perception towards concerns arising from the use of Flying cars	Concerns arising from: ownership cost, safety, operational complexities, operational noise, security and personal information privacy.	4-point Likert scale – not concerned at all to very concerned.
Perception towards benefits resulting from the use of flying cars	Benefits in terms of: reduction in number and severity of crashes, lower and more reliable travel time, likelihood of fuel, maintenance and insurance cost reduction, likelihood of greenhouse gas emission.	4-point Likert scale - very unlikely to very likely.
Willingness to use Flying cars for different trip purposes	Likelihood of using flying cars for different trip purposes: Work, education, entertainment, sports, shopping, trip to airport, trips to/from downtown areas. Likelihood of using flying cars for different trip lengths: short (< 50 miles), medium (50–100 miles), long (100–300 miles), very long (> 300 miles). Likelihood of using flying cars for trips made during different times of day: morning trips (6AM-12PM), afternoon trips (12PM–6PM), evening trips (6PM–12AM), night trips (12AM–6AM).	4-point Likert scale - very unlikely to very likely.
Likelihood of considering residence relocation, given the widespread use of flying cars	Likelihood of relocating to: city centre, urban area (outside city centre), suburban area, rural area, and not relocating at all.	4-point Likert scale - very unlikely to very likely.
Perception towards potential measures to improve safety and security of flying cars	Potential of proposed measures in improving security of flying car operation: use existing FAA regulation for air traffic control, establish air-road police enforcement (with flying police cars), detail profiling and background checking of flying car owners and operators, Establishing no-fly zones near sensitive locations (military bases, power plants, government establishments).	4-point Likert scale - very unlikely to very likely.
Driving behaviour and habits	Questions aimed at capturing past driving history, and self-perceived driving attitudes.	Multiple choice, open ended.
Socio-demographics	Questions aimed at collecting the socio-demographic characteristics of the respondents.	Multiple choice, open ended.

The dataset contains responses from 692 respondents, 584 of which are collected from the United States, 50 from India, and the remaining 58 are from seventeen different countries. These seventeen countries are: Australia; Canada; Dominican Republic; Greece; Iran; Nepal; New Zealand; Nigeria; Oman; Qatar; Saudi Arabia; Sri Lanka; Switzerland; Thailand; Turkey; United Arab Emirates; and United Kingdom. The socio-demographic characteristics of the respondents are summarized in Table 2. As it can be seen from Table 2, 56.9% of the respondents are male. In terms of marital status, 23.5% of the respondents are married and 69.1% are single. With regard to the educational background of the respondents, 49.1% of the respondents have a college degree and 22.9% a post graduate degree.

**Table 2:** Descriptive statistics of key socio-demographic characteristics of the survey respondents (*N* = 692).

Characteristics	Mean/percentage	Std. Dev.	Min.	Max.
**Gender**				
Male	56.9%	–	–	–
Female	39.8%	–	–	–
Other	0.6%	–	–	–
**Age**	30.432	12.729	16	94
**Marital status**				
Single	69.1%	–	–	–
Married	23.5%	–	–	–
Separated	0.5%	–	–	–
Divorced	2.4%	–	–	–
Other	4.5%	–	–	–
**Education**				
Some high school	1.1%	–	–	–
High school diploma	21.4%	–	–	–
Technical college degree	5.5%	–	–	–
College degree	49.1%	–	–	–
Post graduate degree	22.9%	–	–	–
**Ethnicity**				
African American	3.1%	–	–	–
American Indian	2.1%	–	–	–
Asian	22.6%	–	–	–
Caucasian/White	56.9%	–	–	–
Hispanic/White	3.9%	–	–	–
Hispanic/Non-white	0.6%	–	–	–
Other	4.8%	–	–	–
Did not answer	6.0%	–	–	–

List of the available files in the dataset and corresponding description is presented in Table 3. Full details of the questionnaire and the resulting variables is readily available in Mendeley Data repository.

**Table 3:** List of available files in the dataset.

File name	Description	Format
Dataset	Complete dataset consisting of responses from 692 survey participants. The first row represents the variable header, identifying the coded variables (starting from X0 to X112). The remaining rows represent responses from each survey participant.	.csv
Dataset Description	Complete description of the dataset. It consists of the questions (as presented to the survey participants), and how each response is numerically coded within the dataset.	.pdf

## 2 Experimental Design, Materials and Methods

The survey was conducted through the online survey administration platform “SurveyMonkey”, and was disseminated by 35 students and employees from the University at Buffalo. The survey was open throughout the month of March in 2017. The 35 survey collectors gathered responses from 692 survey participants.

The questionnaire was organized in a segmented fashion. In the first section, the respondents were asked about their level of familiarity with modern vehicle safety features, and whether they currently own or previously owned any vehicles with such features. In the second section, the respondents were asked about their willingness to use flying cars, and how much they are willing to pay to purchase a flying car once they become available. They were also asked whether they are interested about using flying taxi services, and how much they would be willing to pay for such service as compared to existing ground-based ridesharing services such as Uber and Lyft. The next sets of questions focused on concerns and benefits that may arise from the future use of flying cars. This was followed by several questions focusing on the respondents’ willingness to use flying cars for different trip purposes, and questions inquiring whether the respondents would be willing to consider relocating their residences when flying cars become available for use. Then the respondents were asked about their perception towards a number of hypothetical security measures to improve safety and security of flying car operations. Finally, the survey concluded with several driving and travel behaviour and habit related questions, followed by socio-demographic characteristics of the respondents.

To capture public perceptions, willingness to use, willingness to pay for emerging transportation technologies through a survey-based framework, the Likert scale is widely accepted in the literature [1], [2], [3], [4]. To that end, a 4-point Likert scale was adopted in this survey. A summary of the questions and the used scales are presented in Table 1. Original research based on this dataset include: [5], [6], [7], [8], [9], [10].

## Ethics Statements

Prior to the survey, informed consent was obtained from all respondents, and they were also notified that their participation in the survey is voluntary, they are free to pause or leave the survey at any point. The respondents were also ensured that their responses would be completely anonymous.

## CRediT authorship contribution statement

**Ugur Eker:** Conceptualization, Methodology, Investigation, Formal analysis. **Grigorios Fountas:** Conceptualization, Methodology, Investigation, Formal analysis, Writing – review & editing. **Sheikh Shahriar Ahmed:** Formal analysis, Writing – original draft. **Panagiotis Ch. Anastasopoulos:** Conceptualization, Methodology, Writing – review & editing, Supervision.

## Declaration of Competing Interest

The authors declare that they have no known competing financial interests or personal relationships that could have appeared to influence the work reported in this paper.

## References

[1] Kyriakidis M., Happee R., de Winter J.C.F. (2015). Public opinion on automated driving: results of an international questionnaire among 5000 respondents. Transp. Res. Part F Traffic Psychol. Behav..

[2] Payre W., Cestac J., Delhomme P. (2014). Intention to use a fully automated car: attitudes and a priori acceptability. Transp. Res. Part F Traffic Psychol. Behav..

[3] Becker F., Axhausen K.W. (2017). Literature review on surveys investigating the acceptance of automated vehicles. Transportation.

[4] Gkartzonikas C., Gkritza K. (2019). What have we learned? A review of stated preference and choice studies on autonomous vehicles. Transp. Res. Part C Emerg. Technol..

[5] Ahmed S.S., Fountas G., Eker U., Anastasopoulos P.C. (2021). Are we willing to relocate with the future introduction of flying cars? An exploratory empirical analysis of public perceptions in the United States. Transp. A Transp. Sci..

[6] Ahmed S.S., Fountas G., Eker U., Still S.E., Anastasopoulos P.C. (2021). An exploratory empirical analysis of willingness to hire and pay for flying taxis and shared flying car services. J. Air Trans. Manag..

[7] Eker U., Ahmed S.S., Fountas G., Anastasopoulos P.C. (2019). An exploratory investigation of public perceptions towards safety and security from the future use of flying cars in the United States. Anal. Methods Accid. Res..

[8] Eker U., Fountas G., Anastasopoulos P.C. (2020). An exploratory empirical analysis of willingness to pay for and use flying cars. Aerosp. Sci. Technol..

[9] Eker U., Fountas G., Anastasopoulos P.C., Still S.E. (2020). An exploratory investigation of public perceptions towards key benefits and concerns from the future use of flying cars. Travel Behav. Soc..

[10] Ahmed S.S., Hulme K., Fountas G., Eker U., Benedyk I., Still S., Anastasopoulos P.C. (2020). The flying car–challenges and strategies towards future adoption. Front. Built Environ..

